# 

**Published:** 1881-08

**Authors:** 


CONTENTS.

| | PAGE. | | PAGE. |
|---|---|---|---|
| Position of the Body | 285 | Lead Colic | 306 |
| Baths and Bathing | 287 | The Teeth | 307 |
| Glucose | 291 | Lives of Medical Men | 308 |
| Medicinal Qualities of Buttermilk | 291 | Metamorphoses | 310 |
| Good-night (poetry) | 292 | Catarrh | 311 |
| Moonlighting | 293 | Some of the Great Bridges | 313 |
| Color of Lightning | 294 | Paper Plates | 314 |
| Seth Green's Spider Story | 295 | Fashionable Freaks | 315 |
| One and Then Another (poetry) | 295 | How to Eat Lemons | 316 |
| The Vagabond Sage | 296 | How to Behave at a Party | 318 |
| Health Maxims | 297 | Silk Worms at Work | 319 |
| A Happy Home | 304 | Anti-Fat | 320 |

